# Nucleic Acid Oxidation in Human Health and Disease

**DOI:** 10.1155/2013/368651

**Published:** 2013-12-25

**Authors:** Mu-Rong Chao, Pavel Rossner, Siamak Haghdoost, Hueiwang Anna Jeng, Chiung-Wen Hu

**Affiliations:** ^1^Department of Occupational Safety and Health, Chung Shan Medical University, Taichung 402, Taiwan; ^2^Department of Occupational Medicine, Chung Shan Medical University Hospital, Taichung 402, Taiwan; ^3^Laboratory of Genetic Ecotoxicology, Institute of Experimental Medicine, Academy of Sciences of the Czech Republic, Videnska 1083, 142 20 Prague, Czech Republic; ^4^Centre for Radiation Protection Research, Department of Molecular Bioscience, Wenner-Gren Institute, Stockholm University, 10691 Stockholm, Sweden; ^5^School of Community and Environmental Health, College of Health Sciences, Old Dominion University, Norfolk, VA 23529, USA; ^6^Department of Public Health, Chung Shan Medical University, Taichung 402, Taiwan

Growing scientific evidence suggests that oxidative stress plays an important role in human health and disease. Under oxidative stress, the excess levels of reactive oxygen species (ROS) may lead to modification of cellular nucleic acids. Oxidatively damaged DNA has been recognized in association with the development of aging, cancer, and some degenerative diseases. The topics covered in this special edition give some insight into how oxidatively damaged DNA is involved in human disease and its health impacts. This special issue contains five papers. Two papers are related to human disease, including Keshan disease and chronic kidney disease, while two papers cover the underlying mechanisms of inflammation-related carcinogenesis and the association between DNA damage and antioxidant capacity in humans. Finally, a paper summarizes the literature regarding the effect of phytoagents on nucleic acid oxidation in cancer cells.

Keshan disease (KD) is an endemic cardiomyopathy of unknown etiology affecting inhabitants of a narrow belt between Northeast China and Southwest China. This region is known for low levels of selenium in the environment as well as in food. In an original research article entitled “*Oxidative stress is involved in the pathogenesis of Keshan disease (an endemic dilated cardiomyopathy) in China,*” J. Pei et al. demonstrate that oxidative stress in the myocardium may play a crucial role in KD. The authors found elevated 8-oxodG levels in myocardial nuclei of the KD patients. Moreover, the expression of glutathione peroxidase 1 and thioredoxin reductases 1 (selenoproteins) was important in the antioxidant system of the organism and higher in the control group than in patients suffering from KD.

In a review article entitled “*Oxidative stress and nucleic acid oxidation in patients with chronic kidney disease,*” C.-C. Sung et al. provide a systematic review of the role of oxidative stress in chronic kidney disease (CKD). It begins with the mechanisms of radical production, antioxidant defense, pathogenesis, and recent biomarkers of oxidative stress in CKD patients. The authors also summarized and evaluated the potential benefit of antioxidant therapies in CKD patients, although their value as useful therapeutic tools is being tested and future studies are necessary to validate their prospective beneficial effects on CKD.

Infectious agents (e.g., parasites and viruses) have been identified as carcinogenic to humans (IARC, group 1). The underlying mechanism of their carcinogenicity includes inflammation accompanied by generation of reactive oxygen (ROS) and nitrogen species (RNS). In a review article entitled “*DNA damage in inflammation-related carcinogenesis and cancer stem cells*,” S. Ohnishi et al. propose a model by which chronic inflammation by infectious agents induces generation of cancer stem cells. The authors suggest that tissue injury caused by ROS/RNS may activate progenitor/stem cells for regeneration. Oxidative stress may then cause multiple mutations which may result in the formation of mutant stem cells and cancer stem cells thus leading to carcinogenesis.

In an original research article entitled “*Is the oxidative DNA damage level of human lymphocyte correlated with the antioxidant capacity of serum or the base excision repair activity of lymphocyte?*,” Y.-C. Tsai et al. investigate the individual levels of oxidative DNA base damage in the human lymphocyte from different donors (healthy and infected patients) and its correlation with total antioxidant levels in the serum and the DNA base excision repair capacity. The most important finding is that antioxidant serum levels may not change the steady state levels of the oxidative base damage in the DNA. One should also consider that antioxidants in our body protect us in acute and highly stressful situations when we have high levels of ROS in a short time period rather than decrease the steady state levels.

In a review article entitled “*Phytoagents for cancer management: regulation of nucleic acid oxidation, ROS, and related mechanisms*,” W.-L. Lee et al. present a detailed review of the role of phytoagents as redox regulators of nucleic acid oxidation in carcinogenesis. On the concept of genetic heterogeneity caused by nucleic acid oxidation as a major driving force of cancer progression, the authors highlight how and why phytoagents induce or prevent oxidative stress and their potential use in cancer prevention or therapy.

